# Judicialization of medicines: effectiveness of rights or break in public policies?

**DOI:** 10.11606/s1518-8787.2020054002301

**Published:** 2020-11-27

**Authors:** Yonara Monique da Costa Oliveira, Bárbara Suellen Fonseca Braga, Andrezza Duarte Farias, Sylvia Patrícia Dantas Pereira, Maria Angela Fernandes Ferreira

**Affiliations:** I Universidade Federal de Campina Grande Centro de Educação e Saúde Curso de Bacharelado em Farmácia CuitéPB Brasil Universidade Federal de Campina Grande. Centro de Educação e Saúde. Curso de Bacharelado em Farmácia. Cuité, PB, Brasil; II Universidade Federal do Rio Grande do Norte Centro de Ciências da Saúde NatalRN Brasil Universidade Federal do Rio Grande do Norte. Centro de Ciências da Saúde. Programa de Pós-Graduação em Saúde Coletiva. Natal, RN, Brasil; III Universidade Federal do Rio Grande do Norte Centro de Ciências da Saúde Departamento de Odontologia NatalRN Brasil Universidade Federal do Rio Grande do Norte. Centro de Ciências da Saúde. Departamento de Odontologia. Natal, RN, Brasil

**Keywords:** Access to Essential Medicines and Health Technologies, Health's Judicialization, legislation & jurisprudence, National Policy of Pharmaceutical Assistance, Right to Health

## Abstract

**OBJECTIVE:**

To analyze whether lawsuits for medicines filed against the state of Rio Grande do Norte agree with medical-sanitary and pharmaceutical assistance management criteria established by the public policies of access to medicines in force in Brazil.

**METHODS:**

This is a descriptive and retrospective study of the individual lawsuits that claimed medicines in the state of Rio Grande do Norte between 2013 and 2017. Information was collected from the procedural documents on the requested medicines, the diagnoses referred and the origin of the medical prescription, in order to analyze medical-sanitary and pharmaceutical assistance management characteristics.

**RESULTS:**

We analyzed 987 lawsuits, which requested 1,517 medications. Of these, 60.7% were not part of the National List of Essential Medicines, and, in 75% of the cases, there was a therapeutic alternative in the Brazilian Unified Health System. In 13.6% of the actions, at least one drug was prescribed for off-label use. Prescribers of philanthropic and private services often request medicines not covered by the pharmaceutical care policy. Even judicialized drugs that are part of the national list are constantly requested for non-standard indications.

**CONCLUSIONS:**

Court decisions for the supply of medicines violate health rules and make it difficult to manage pharmaceutical assistance, which may weaken the implementation of these policies.

## INTRODUCTION

Medicines are fundamental to health systems, and their use involves a conflicting social context: they are an essential good, but also a product with high market value. Given their importance, in many countries, access to essential medicines is an integral part of human rights ^1^ .

In Brazil, integral therapeutic care, including pharmaceutical assistance, is a right guaranteed to all citizens, being part of the list of services provided by the Unified Health System (SUS). After implementing the SUS as public health policy in Brazil, pharmaceutical assistance (PA) was formally included by ordinance GM/MS no. 3.916/1998, which instituted the *Política Nacional de Medicamentos* (PNM – National Medicine Policy), aiming to ensure universal access to safe, effective, quality medicines at the lowest possible price for all. Subsequently, PA was ratified as a public health policy by resolution no. 338 of 2004 of the *Conselho Nacional de Saúde* (CNS – National Health Council), which instituted the *Política Nacional de Assistência Farmacêutica* (PNAF – National Pharmaceutical Assistance Policy) ^2^ .

Among the priority guidelines of PNM, the adoption of *Relação Nacional de Medicamentos Essenciais* (Rename – National List of Essential Medicines), permanently revised, is considered a guiding point of medicine policy, to guide administration and rational use. Currently, the PA in SUS is organized in three components: basic, composed of medicines to treat the most prevalent diseases in primary health care; strategic, for medicines for endemics and diseases of epidemiological importance; and specialized, for medications with use described in the *Protocolos Clínicos e Diretrizes Terapêuticas* (PCDT – Clinical Protocols and Therapeutic Guidelines) ^3^

Despite the advances observed after more than 20 years of PNM, new challenges have been imposed, among them the phenomenon of judicialization in access to medicines. Protected by the constitutional right to health, individuals resort to the Court to have access to medications, both those already incorporated into SUS ^4^ and those that are not yet available by it ^5^ .

Some authors also state that judicialization is a strategy of pressure to incorporate new technologies ^6^^,^^7^ . Therefore, to ensure health, and thus the dignity of the human person, sanitary aspects that involve lawsuits for the supply of medicine deserve attention ^8^ .

In this context, the objective of this study was to analyze whether the individual lawsuits requesting medicines, filed against the state of Rio Grande do Norte, agree with the medical-sanitary and pharmaceutical assistance administration criteria established by public policies of access to medicines in force in the country.

## METHODS

### Study Design

This is a descriptive and retrospective study whose unit of analysis was the individual lawsuit requesting medicines filed by the citizen against the state of Rio Grande do Norte.

The research included processes pending in the first instance, with distribution date between January 2013 and December 2017. The study period has as initial milestone the year from which the *Secretaria de Estado de Saúde Pública do Rio Grande do Norte* (Sesap/RN – State Department of Public Health of Rio Grande do Norte) began to systematically organize lawsuits requesting medications, with 2017 as the final year, thus analyzing a five-year period. We excluded from the database lawsuits that were in legal confidentiality, public civil actions and those that did not request medicines.

### Variables

The study variables were selected from the *Manual de Indicadores de Avaliação e Monitoramento das Demandas Judiciais por Medicamentos*^9^ . To meet the objective of the study, we chose variables from lawsuits that evaluated medical-sanitary and pharmaceutical assistance management characteristics, which were: name of the drug(s) requested, presence of the drug in Rename, proportion of drugs demanded by pharmaceutical assistance financing block, proportion of drugs demanded with therapeutic alternative in SUS, proportion of lawsuits with at least one medication prescribed for off-label use, origin of the medical prescription (public, private or philanthropic service), agreement of the drugs demanded and present in Rename with the PCDT.

### Data Collection and Classification

Data regarding lawsuits were obtained in the Judicial Demands Center of the *Unidade Central de Agentes Terapêuticos* (Unicat – Central Unit of Therapeutic Agents). This database provided to the researchers the number of the lawsuit, the name of the author of the action and the medicine requested. From this information, we consulted the case files available in the procedural management systems of the *Tribunal de Justiça do Rio Grande do Norte* (TJRN – Court of Justice of Rio Grande do Norte) and the *Justiça Federal no Rio Grande do Norte* (JFRN – Federal Court of Rio Grande do Norte) in order to collect detailed information on the lawsuits and build a database with the variables of interest. For this purpose, a semi-structured questionnaire was constructed, using the Excel software (Microsoft, 2013).

After collection, we reviewed the data and classified the medicines using the Anatomical Therapeutic Chemical System (ATC) The presence of the medicine in official public lists was verified by consulting the Rename 2014, which was in force for most of the study period. The same relation was consulted to assess the presence of therapeutic alternative in SUS, considering that a drug from the same pharmacological subgroup by ATC classification for the same therapeutic indication is interchangeable.

Off-label use was determined after analyzing the diagnostic indication in the records of the lawsuit, and consulting the medicine leaflet, using as source the National Sanitary Surveillance Agency (ANVISA)ᵃ. We considered off-label a medicine prescribed for an indication other than that approved by health autorities ^9^ . Medicines judicially demanded that were part of the *Componente Especializado da Assistência Farmacêutica* (CEAF – Specialized Component of Pharmaceutical Care) were analyzed considering if they agreed with therapeutic indication with the PCDT in force. Figure 1 summarizes data collection and analysis process.

**Figure:**
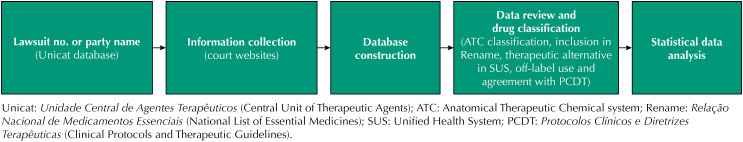
Flowchart of data collection and classification.

### Statistical Analyses

For data analysis, the SPSS software was used (version 20). Continuous variables were categorized and showed as absolute and relative frequency. To compare categorical variables, we used the Pearson chi-square test, with a 5% significance level.

### Ethical Considerations

This study is the result of a research project entitled *Análise das demandas judiciais por medicamentos no Rio Grande do Norte* , which was submitted and approved by the Research Ethics Committee of the University Hospital Onofre Lopes, with opinion no. 2.404.850.

## RESULTS

We identified 1,635 lawsuits, of which 987 fell within the inclusion criteria and were analyzed in the investigation. A total of 1,517 medicines were requested, being 328 different items, with an average request of 1.55 (standard deviation of 1.40) drugs per legal action.

The analyses of medical-sanitary and pharmaceutical assistance management variables showed that most of the medicines judicially requested are not yet incorporated into SUS. Of those which were requested and included in the Rename, the ones belonging to CEAF were most frequent. We observed that there is a high proportion of medicines not included in the Rename with therapeutic alternative in SUS. Of the 987 analyzed lawsuits, we identified that, in 13.6%, there was at least one drug prescribed for an indication not described in the leaflet, that is, an off-label use ( Table 1 ).

**Table 1:** Medical-sanitary and pharmaceutical assistance management characteristics of lawsuits requesting medicines. Rio Grande do Norte, 2013–2017.

		n	%
Presence in Rename (n = 1,517)			
	No	922	60.7
	Yes	596	39.3
Pharmaceutical assistance component (n = 596)			
	CBAF	192	32.2
	CEAF	402	67.4
	CESAF	2	0.4
Proportion of drugs demanded outside Rename with alternative in SUS (n = 922)		692	75.0
Proportion of off-label use (n = 987)		134	13.6

Rename: *Relação Nacional de Medicamentos Essenciais* (National List of Essential Medicines); CBAF: *Componente Básico da Assistência Farmacêutica* (Basic Component of Pharmaceutical Assistance); CEAF: *Componente Especializado da Assistência Farmacêutica* (Specialized Component of Pharmaceutical Assistance); CESAF: *Componente Estratégico da Assistência Farmacêutica* (Strategic Component of Pharmaceutical Assistance); SUS: Unified Health System

Source: Research data, 2019.

When evaluating the relation between type of drug requested according to the ATC classification and its inclusion in Rename, we observed a significant association (p<0.001), showing that the proportion of requested medicines included in Rename varies according to the therapeutic subgroup. Drugs that act on the respiratory, musculoskeletal and digestive systems and metabolism, as well as antineoplastic agents and immunomodulators, have higher proportion not included in Rename, while medications that act in the cardiovascular and nervous systems and systemic hormonal preparations have a higher proportion of incorporation in the national list ( Table 2 ). When analyzing the association between origin of the prescription and inclusion in Rename, we noticed that prescriptions from philanthropic and private services request more medicines outside Rename than those from public services ( Table 2 ).

**Table 2:** Classification of medicines and origin of medical prescription of drugs requested in lawsuits according to the inclusion in Rename. Rio Grande do Norte, 2013–2017.

		Inclusion in Rename		Total
		No	Yes	
1^st^ ATC level a	
	A- Digestive system and metabolism	247 (80.7%)	59 (19.3%)	306
	B- Blood and blood-forming organs	101 (72.7%)	38 (27.3%)	139
	C- Cardiovascular system	90 (45.5%)	108 (54.5%)	198
	H- Systemic hormonal preparations	54 (38.3%)	87 (61.7%)	141
	L- Antineoplastic and immunomodulating agents	185 (57.3%)	138 (42.7%)	323
	M- Musculoskeletal system	41 (80.4%)	10 (19.6%)	51
	N- Nervous system	79 (45.4%)	95 (54.6%)	174
	R- Respiratory system	82 (88.2%)	11 (11.8%)	93
	Others	43 (46.2%)	50 (53.8%)	93
	Total	922 (60.7%)	596 (39.1%)	1,517
Origin of medical prescription b				
	Private services	224 (59.6%)	152 (40.4%)	376
	Public services	123 (53.2%)	108 (46.7%)	231
	Philanthropic services	61 (75.3%)	20 (24.7%)	81
	Total	408 (59.3%)	208 (30.2%)	688

Rename: *Relação Nacional de Medicamentos Essenciais* (National List of Essential Medicines); ATC: Anatomical Therapeutic Chemical System.

a Chi-square test: p < 0.001.

b Chi-square test: p < 0.002.

Source: Research data, 2019.

In Table 3 , the 10 most frequently requested drugs are listed according to their inclusion in Rename, therapeutic alternative, and, in the case of those that are included in official lists, agreement with the current PCDT. Of the drugs not incorporated to the pharmaceutical care policy, insulin glargine, tiotropium bromide, fast-acting insulin analogues (lispro and aspart) and osteoporosis treatment drugs (teriparatide and denosumab) led the requests; there was no alternative in SUS for only 2 of the 10 most requested medicines (bevacizumab for use as an antineoplastic agent and for intravitreal use).

**Table 3:** Most requested drugs in lawsuits according to the inclusion in Rename, therapeutic alternative in SUS and agreement with PCDT. Rio Grande do Norte, 2013–2017.

Drugs not included in Rename (n = 922)	n	%	Therapeutic alternative in SUS	Medicines included in Rename (n = 596)	n	%	Agreement with PCDT (%)
Insulin glargine	74	8.0	Y	Somatropin	53	8.9	100.0
Tiotropium bromide	58	6.3	Y	Rituximab	25	4.2	8.0
Enoxaparin	57	6.2	Y	Azathioprine	21	3.7	90.5
Insulin lispro	32	3.5	Y	Mycophenolate mofetil	18	3.1	0.0
Teriparatide	30	3.3	Y	Clopidogrel	16	2.7	12.5
Bevacizumab (intravitreal)	25	2.7	N	Infliximab	14	2.3	21.4
Insulin aspart	24	2.6	Y	Acetylsalicylic acid	13	2.2	100.0
Bevacizumab	21	2.3	N	Goserreline	13	2.2	23.1
Denosumab	21	2.3	Y	Deferasirox	12	2.0	25.0
Rivaroxabane	19	2.1	Y	Mesalazine	12	2.0	83.3

Rename: *Relação Nacional de Medicamentos Essenciais* (National List of Essential Medicines); SUS: Unified Health System; PCDT: *Protocolos Clínicos e Diretrizes Terapêuticas* (Clinical Protocols and Therapeutic Guidelines); Y: yes; N: no

Source: Research data, 2019.

Finally, of the requested drugs contemplated in the policy, Somatropin was the most requested, with the indication of the request agreeing 100% with the clinical guidelines of SUS, a situation similar to requests of azatioprine and mesalazine. The other drugs, although included in the policy, were frequently requested for non-standard indications in the PCDT, with mycophenolate mofetil standing out, which was judicially requested in 18 actions, none of them for the standardized indication by SUS ( Table 3 ).

## DISCUSSION

In this study, we observed that most of the drugs requested from Court are not incorporated into SUS, although there is a therapeutic alternative available. There is an association between the claimed class of drugs and its presence in Rename, with a low adherence of prescribers to drugs included in the components of pharmaceutical assistance and a predominance of prescribed drugs not included in Rename by doctors linked to philanthropic and private institutions. Even in the case of drugs in the list, we observed prescriptions that disagreed with the indication provided in PCDT.

Empirical studies that evaluated the type of drug required show that there is no national standard. There are investigations with predominance of requests for medicines from public policy ^4^^,^^5^^,^^10^^,^^11^ ; however, most of the empirical data are similar to ours, with most of the judicialized drugs not included in Rename ^12^^–^^16^ .

A hypothesis that justifies this heterogeneous panorama is that health judicialization show relevant regional differences that are reflected in health care and justice systems. Therefore, in states in which the largest number of lawsuits is for medicines included in the national policy, there may be failures in the management of pharmaceutical care, while in those in which requests are mainly for drugs outside the official lists, the policy works well, and only what is not provided to the citizen because it is still not available in SUS reaches the court ^17^ .

Another hypothesis for the judicialization of medicines not yet available in SUS is that there is a delay in the incorporation of technologies, as well as a lack of technical criteria and transparency in the incorporation process ^18^ . However, an analysis that compared the Rename editions published from 2000 to 2014 observed an increased number of medicines in the editions published after 2012, without the expected decrease in judicialization and in the pressure for the technological incorporation of innovative products, which, on the contrary, have increased in recent years ^19^ . Rename suffered alterations in its elaboration process, moving from a list based on the concept of essentiality in the 1998 to 2010 editions, to a positive relation of drugs covered by the SUS from 2012, with the publication of law no.12.401/2011 and decree 7.508/2011, which regulated the concept of integrality in the SUS and brought changes to the technology incorporation process ^7^^,^^19^ .

Our data showed that most of the claimed drugs outside Rename have a therapeutic alternative in SUS. For example, one of the most requested drugs was tiotropium bromide, indicated for treating asthma or chronic obstructive pulmonary disease (COPD), which has the association of formoterol and budesonide as therapeutic alternative in SUS, belonging to CEAF. Previous studies that evaluated the proportion of requested drugs that have therapeutic alternative in SUS showed prevalence ranging from 41.7% to 96.0%. These data show that the SUS administration is not completely omitted, since it provides treatment options for the diseases that justify the judicial requests ^20^ .

Some classes of medicines are preferred targets of requests for non-incorporated drugs, although they do not treat the most prevalent diseases in the population. An analysis of the applications for incorporation to Rename from 2012 to 2016 showed that medications in the anti-infectious classes for systemic use to treat HIV/AIDS and hepatitis C, and the ones indicated for musculoskeletal disorders, neoplams, mental and behavioral disorders, and respiratory diseases accounted for 64.5% of the incorporated drugs. These data show that there is an increase in the availability of medicines by SUS, including classes that are usually demanded judicially, which corroborates our findings ^21^ .

By abandoning the concept of essentiality, based on the epidemiological demands of the population, Rename has become more susceptible to political and social pressures to incorporate new technologies, which do not always show therapeutic superiority. This is a worrying fact that opposes the guidelines established by the PNM, since it compromises PA management and the adherence of medical professionals to the SUS lists ^18^^,^^19^ .

Another priority guideline established by PNM is rational use and safety of medicines, being the off-label prescription an indication of the lack of efficacy and safety in use. Our data show that the most prescribed medicine for off-label use was intravitreal bevacizumab, with 35 requests for various clinical indications, with diabetic retinopathy, glaucoma and age-related macular degeneration (AMD) standing out.

Bevacizumab is registered in ANVISA with indication for the treatment of cancer, but it has been used off-label for AMD. In view of intense judicialization and the lack of therapeutic option in SUS, in 2016, ANVISA authorized its exceptional and temporary use for three years for the treatment of AMD, according to rules established in a protocol that provides intravitreal use only for AMD (code H35.3 of the 10th revision of the International Classification of Diseases) ^22^ . However, we found that, of the 35 judicial requests for this drug, only five had this indication, evidencing that the SUS has sought to fill gaps in its policies, however, new uses and indications end up continuously fueling judicialization.

One of the protagonists of health judicialization are medical professionals. Studies on the topic have pointed out the relations between prescribers, pharmaceutical industry and lawyers who act in a coordinated manner, generating judicial demand for medicines and, thus, pressing their incorporation ^6^^,^^23^^,^^24^ . In our study, we observed that doctors from philanthropic institutions and private health services prescribe more drugs not included in Rename than doctors from SUS, despite the fact that all prescribers predominantly request non-standard drugs.

This shows that the authors from private and philanthropic services resort to the Court to have access, by SUS, mainly to medicines not incorporated by the system. It is noteworthy that, in our sample, the main philanthropic service that generated lawsuits was an oncological hospital affiliated to SUS and that, therefore, already receives funding through *Procedimento de Alta Complexidade em Oncologia* (Authorization of High Complexity Procedure in Oncology). Empirical studies show that the main judicial criterion for a favorable decision to the litigant is the evidence shown by the plaintiff, usually based on a medical report or prescription ^25^^,^^26^ .

Therefore, caution is necessary, above all, in relation to the prescription of medicines non included in the official lists of drugs or in non-compliance with officially established clinical protocols. It is necessary to question whether the intended treatment has scientific evidence and if it is possible to replace it with a therapeutic alternative available in the public health network, because, otherwise, the harmful effects of fragmented individual decisions will undermine public policies, which make priority choices for the allocation of scarce resources. Ignoring public policies ultimately strengthens discriminatory situations and unsustainable medical technologies from the scientific point of view ^26^^,^^27^ .

When analyzing the most requested drugs not available in SUS, we see the predominance of requests for both short and long-acting insulin analogues. Empirical studies carried out in other states, such as São Paulo and Bahia, also found insulin analogue as the most requested drugs not included in Rename ^12^^,^^28^^,^^29^ . Of the 10 most requested drugs, only bevacizumab for intravitreal use and for cancer treatment does not have an alternative available in the SUS.

It is also interesting to observe the dynamics between judicialization and incorporation of technologies into SUS. Five of the ten drugs most judicially requested to the state in the study period, including some with an opinion contrary to the incorporation into the SUS, were recently incorporated, between 2016 and 2019. The case of insulin analogue is didactic for this relation between judicialization and incorporation, since, after two negative stances on incorporation in 2014, the *Comissão Nacional de Incorporação de Tecnologias* no SUS (Conitec – National Commission for Incorporation of Technologies in SUS), recommended the incorporation of fast-acting insulins by SUS for the treatment of type 1 diabetes mellitus (DM1) in 2017, and, in March 2019, a new, favorable opinion was issued in favor of long-acting insulin analogue for DM1, even without new scientific evidence of superiority. The opinion described the “lawsuits of these drugs” as an issue to be analyzed ^22^ .

Other drugs targeted by lawsuits recently incorporated were enoxaparin for the prevention of thrombophilia in pregnant women and the already described intravitreal bevacizumab, with exceptional use approved for three years. The others were evaluated by Conitec, which opposed the incorporation since they do not show a superior efficacy to technologies already available in SUS. Only denosumab has no record of evaluation by Conitec to date ^22^ .

Although most of the drugs judicially requested are not included in Rename, a considerable percentage of these medicines already incorporated by the policy was also requested, most being from CEAF. The Judiciary search for medicines of this component may mean failures in its management, as well as attempts to circumvent the legally established criteria. When analyzing the agreement between the requested drug and the clinical indication described in the medical report with the current PDCT, we observed possible failures in the management of pharmaceutical care in the request for the drugs somatropin, azathioprine, acetylsalicylic acid and mesalazine, since the vast majority of requests agreed with protocols. For the other drugs requested, the highest prevalence was for clinical indications non-standard in official protocols, most for off-label uses. Mycophenolate mofetil stands out, for which none of the clinical indications described in the lawsuits agreed with the PCDT, and in 12 of the 18 solicitations, it was prescribed for off-label use. We observed that lawsuits usually lack technical evidentiary criteria, being instructed with medical report and prescription, rarely with the presence of examinations, and have made the judicial route a faster way with higher success rates in obtaining the medicine requested, since these documents would be insufficient in many cases to ensure access by the SUS ^8^^,^^26^ .

This study has limitations, since we portray a local reality; however, considering the lack of multicenter studies and of a national panorama of judicialization in access to medicines, we considered that local research is necessary to deepen the knowledge on the phenomenon. In addition, the study was carried out from data consulted in procedural documents available in the court management systems, by the information obtained in the Sesap/RN, being a convenience sample, since it there is no specific management system for these lawsuits.

From the data analysis, it may be concluded that, in many cases, the Court generates breaking points in the existing public policy, going over criteria that were elaborated to safeguard the health of the population and a more efficient management of public resources. In this sense, in order to ensure the right to health, sanitary rules are violated, and the management of pharmaceutical assistance is hampered. We emphasize that the supply of a drug does not necessarily translate into a guarantee of the health of the individual. After the judicial decision, the Court or the health services do not officially monitor or verify the conditions of use, the evolution or the achievement of the therapeutic goals intended by the prescriber; thus, the sanitary outcomes for the health of the patients is unknown. Therefore, new investigations should be directed towards this analysis.
