# Supplementary material for: Judicialization of medicines: effectiveness of rights or break in public policies?
Source: Rev Saude Publica. 2020 Nov 27;54:130. doi: 10.11606/s1518-8787.2020054002301 (PMC7703526; doi:10.11606/s1518-8787.2020054002301)
Supplement: Translation

# Translation: Judicialização de medicamentos: efetivação de direitos ou ruptura das políticas públicas?

**Authors:** Yonara Monique da Costa Oliveira, Bárbara Suellen Fonseca Braga, Andrezza Duarte Farias, Sylvia Patrícia Dantas Pereira, Maria Angela Fernandes Ferreira

**Affiliations:** I CuitéPB Brasil Universidade Federal de Campina Grande. Centro de Educação e Saúde. Curso de Bacharelado em Farmácia. Cuité, PB, Brasil; II NatalRN Brasil Universidade Federal do Rio Grande do Norte. Centro de Ciências da Saúde. Programa de Pós-Graduação em Saúde Coletiva. Natal, RN, Brasil; III NatalRN Brasil Universidade Federal do Rio Grande do Norte. Centro de Ciências da Saúde. Departamento de Odontologia. Natal, RN, Brasil

**Keywords:** Acesso a Medicamentos Essenciais e Tecnologias em Saúde, Judicialização da Saúde, legislação & jurisprudência, Política Nacional de Assistência Farmacêutica, Direito à Saúde

## Abstract

**OBJETIVO:**

Analisar se as demandas judiciais por medicamentos movidas contra o estado do Rio Grande do Norte estão de acordo com critérios médico-sanitários e de gestão da assistência farmacêutica estabelecidos pelas políticas públicas de acesso a medicamentos vigentes no Brasil.

**MÉTODOS:**

Foi conduzido um estudo descritivo e retrospectivo das ações judiciais individuais que pleiteavam medicamentos no estado do Rio Grande do Norte entre 2013 e 2017. Foram coletadas informações nos autos processuais sobre os medicamentos solicitados, os diagnósticos referidos e a origem da prescrição médica, a fim de se analisar as características médico-sanitárias e de gestão da assistência farmacêutica.

**RESULTADOS:**

Foram analisados 987 processos, em que foram solicitados 1.517 medicamentos. Desses, 60,7% não faziam parte do elenco da Relação Nacional de Medicamentos Essenciais, e em 75% dos casos havia alternativa terapêutica no Sistema Único de Saúde. Em 13,6% das ações, ao menos um medicamento foi prescrito para uso *off label* . Os prescritores dos serviços filantrópicos e privados solicitam frequentemente medicamentos não contemplados pela política de assistência farmacêutica. Mesmo os medicamentos judicializados que fazem parte da relação nacional são constantemente solicitados para indicações não padronizadas.

**CONCLUSÕES:**

As decisões judiciais para o fornecimento de medicamentos violam regras sanitárias e dificultam a gestão da assistência farmacêutica, o que pode enfraquecer a execução dessas políticas.

## INTRODUÇÃO

Os medicamentos são fundamentais para os sistemas de saúde e seu uso envolve um contexto social conflituoso: são um bem essencial, mas também um produto com alto valor de mercado. Dada sua importância, em muitos países, o acesso a medicamentos essenciais é parte integrante dos direitos humanos ^1^ .

No Brasil, a assistência terapêutica integral, inclusive farmacêutica, é um direito garantido a todos os cidadãos, fazendo parte do rol de serviços disponibilizados pelo Sistema Único de Saúde (SUS). Após a institucionalização do SUS como política pública de saúde no Brasil, realizou-se a inclusão formal da assistência farmacêutica (AF) por meio da portaria GM/MS nº 3.916/1998, que instituiu a Política Nacional de Medicamentos (PNM), a qual visa garantir o acesso universal a medicamentos seguros, eficazes, de qualidade e ao menor custo possível para todos. Posteriormente, a AF foi ratificada como uma política pública de saúde por meio da resolução nº 338 de 2004 do Conselho Nacional de Saúde (CNS), que instituiu a Política Nacional de Assistência Farmacêutica (PNAF) ^2^ .

Dentre as diretrizes prioritárias da PNM, a adoção de uma Relação Nacional de Medicamentos Essenciais (Rename), permanentemente revisada, é considerada um ponto norteador da política de medicamentos, para orientar a gestão e o uso racional. Atualmente, a AF no SUS encontra-se organizada em três componentes: básico, composto por medicamentos para o tratamento das doenças mais prevalentes na atenção básica; estratégico, por medicamentos para o tratamento de endemias e doenças com importância epidemiológica; e especializado, por medicamentos cuja utilização está descrita nos Protocolos Clínicos e Diretrizes Terapêuticas (PCDT) ^3^ .

Apesar dos avanços observados após mais de 20 anos da vigência da PNM, novos desafios têm sido impostos, entre eles o fenômeno da judicialização no acesso a medicamentos. Amparados pelo direito constitucional a saúde, os indivíduos recorrem ao Judiciário para ter acesso a medicamentos, tanto aos que já estão incorporados ao SUS ^4^ quanto aos que ainda não são disponibilizados por ele ^5^ .

Alguns autores afirmam, ainda, que a judicialização é uma estratégia de pressão para incorporação de novas tecnologias ^6^^,^^7^ . Portanto, para que se garanta a saúde, e assim a dignidade da pessoa humana, os aspectos sanitários que envolvem as ações judiciais para fornecimento de medicamentos merecem atenção ^8^ .

Nesse contexto, o objetivo do presente estudo foi analisar se as demandas judiciais individuais que solicitam medicamentos, movidas contra o estado do Rio Grande do Norte, estão de acordo com critérios médico-sanitários e de gestão da assistência farmacêutica estabelecidos pelas políticas públicas de acesso a medicamentos vigentes no país.

## MÉTODOS

### Delineamento do Estudo

Trata-se de um estudo descritivo e retrospectivo cuja unidade de análise foi o processo judicial individual de solicitação de medicamentos movido pelo cidadão contra o estado do Rio Grande do Norte.

Foram incluídos na pesquisa processos que tramitavam na primeira instância, com data de distribuição entre janeiro de 2013 e dezembro de 2017. O período de estudo teve como marco inicial o ano a partir do qual a Secretaria de Estado de Saúde Pública do Rio Grande do Norte (Sesap/RN) começou a organizar de forma sistemática os processos solicitando medicamentos, e como final o ano de 2017, realizando-se a análise de um quinquênio. Foram excluídos da análise os processos que tramitavam em segredo de justiça, as ações civis públicas e os que não solicitavam medicamentos.

### Variáveis

As variáveis de estudo foram selecionadas do Manual de Indicadores de Avaliação e Monitoramento das Demandas Judiciais por Medicamentos ^9^ . Para atender ao objetivo da pesquisa, foram eleitas variáveis das demandas judiciais que avaliassem características médico-sanitárias e de gestão da assistência farmacêutica, quais sejam: nome do(s) medicamento(s) solicitado(s), presença do medicamento na Rename, proporção de medicamentos demandados por bloco de financiamento da assistência farmacêutica, proporção de medicamentos demandados com alternativa terapêutica no SUS, proporção de ações judiciais com ao menos um medicamento prescrito para uso *off label,* origem da prescrição médica (serviço público, privado ou filantrópico), concordância dos medicamentos demandados e presentes na Rename com os Protocolos Clínicos e Diretrizes Terapêuticas (PCDT).

### Coleta e Classificação dos Dados

Os dados relativos aos processos judiciais foram obtidos no Núcleo de Demandas Judiciais da Unidade Central de Agentes Terapêuticos (Unicat). Nesse banco fornecido aos pesquisadores, constavam o número do processo, o nome do autor da ação e o medicamento pleiteado. A partir dessas informações, foi realizada consulta aos autos processuais disponíveis nos sistemas de gerenciamento processual do Tribunal de Justiça do Rio Grande do Norte (TJRN) e da Justiça Federal no Rio Grande do Norte (JFRN) a fim de coletar informações detalhadas dos processos e construir o banco com as variáveis de interesse. Para isso, foi construído um questionário eletrônico semiestruturado, com o uso do programa Excel (Microsoft, 2013).

Após a coleta, foi realizada a revisão dos dados e a classificação dos medicamentos utilizando o Sistema Anatômico Terapêutico Químico (código ATC). A presença do medicamento nas listas públicas oficiais foi verificada por consulta à Rename 2014, vigente durante a maior parte do período estudado. A mesma relação foi consultada para avaliar a presença de alternativa terapêutica no SUS, considerando-se intercambiável um medicamento do mesmo subgrupo farmacológico pela classificação ATC para a mesma indicação terapêutica.

O uso *off label* foi determinado após análise da indicação diagnóstica constante nos autos do processo e consulta à bula do medicamento, utilizando como fonte o bulário eletrônico da Agência Nacional de Vigilância Sanitária (Anvisa)ᵃ. Considerou-se uso *off label* quando o medicamento foi prescrito para uma indicação diferente daquela aprovada pela autoridade sanitária ^9^ . Os medicamentos demandados judicialmente que faziam parte do Componente Especializado da Assistência Farmacêutica (CEAF) foram analisados de acordo com a concordância da indicação terapêutica com os PCDT vigentes. A Figura 1 sumariza o processo de coleta e análise dos dados.

**Figura:** Fluxograma da coleta e classificação dos dados.

### Análises Estatísticas

Para a análise dos dados, foi utilizado o programa SPSS (versão 20). As variáveis contínuas foram categorizadas e apresentadas como frequência absoluta e relativa. Para comparação de variáveis categóricas, foi utilizado o teste do qui-quadrado de Pearson, sendo considerado como nível de significância 5%.

### Considerações Éticas

Este trabalho é resultado do projeto de pesquisa intitulado *Análise das demandas judiciais por medicamentos no Rio Grande do Norte* , que foi submetido e aprovado pelo Comitê de Ética em Pesquisa do Hospital Universitário Onofre Lopes, com parecer nº 2.404.850.

## RESULTADOS

Foram identificados 1.635 processos judiciais, dos quais 987 se enquadraram nos critérios de inclusão e foram analisados na pesquisa. Foram solicitados 1.517 medicamentos, sendo 328 itens diferentes, com uma média de pedido de 1,55 (desvio-padrão de 1,40) medicamentos por ação.

A análise das variáveis médico-sanitárias e de gestão da assistência farmacêutica mostrou que a maioria dos medicamentos demandados judicialmente ainda não se encontram incorporados ao SUS. Daqueles que foram solicitados e estão presentes na Rename, os que pertencem ao CEAF foram os mais frequentes. Foi observado que há elevada proporção de medicamentos fora da Rename com alternativa terapêutica no SUS. Dos 987 processos analisados, identificou-se que em 13,6% havia pelo menos um medicamento prescrito para uma indicação não descrita na bula, ou seja, em uso *off label* ( Tabela 1 ).

**Tabela 1:** Características médico-sanitárias e de gestão da assistência farmacêutica das ações solicitando medicamentos. Rio Grande do Norte, 2013–2017.

		n	%
Presença na Rename (n = 1.517)			
	Não	922	60,7
	Sim	596	39,3
Componente da assistência farmacêutica (n = 596)			
	CBAF	192	32,2
	CEAF	402	67,4
	CESAF	2	0,4
Proporção de medicamentos demandados fora da Rename com alternativa no SUS (n = 922)		692	75,0
Proporção de uso *off label* (n = 987)		134	13,6

Rename: Relação Nacional de Medicamentos Essenciais; CBAF: Componente Básico da Assistência Farmacêutica; CEAF: Componente Especializado da Assistência Farmacêutica; CESAF: Componente Estratégico da Assistência Farmacêutica; SUS: Sistema Único de Saúde.

Fonte: Dados da pesquisa, 2019.

Ao avaliar a relação entre o tipo de medicamento solicitado de acordo com a classificação ATC e a sua presença na Rename, foi observada associação significativa (p < 0,001), mostrando que a proporção dos solicitados que constam da Rename varia conforme o subgrupo terapêutico. Medicamentos que atuam no sistema respiratório, sistema musculoesquelético e aparelho digestivo e metabolismo, assim como agentes antineoplásicos e imunomoduladores, têm maior proporção fora da Rename, enquanto os medicamentos que atuam nos sistemas cardiovascular e nervoso e as preparações hormonais sistêmicas têm maior proporção de incorporados na relação nacional ( Tabela 2 ). Analisando a associação entre origem da prescrição e presença na Rename, constatou-se que as prescrições oriundas de serviços filantrópicos e privados de saúde solicitam mais medicamentos fora da Rename do que as originadas em serviços públicos ( Tabela 2 ).

**Tabela 2:** Classificação dos medicamentos e origem da prescrição médica dos medicamentos solicitados nas ações judiciais de acordo com a presença na Rename. Rio Grande do Norte, 2013–2017.

		Presença na Rename		Total
		Não	Sim	
1º nível ATC a	
	A- Sistema digestório e metabolismo	247 (80,7%)	59 (19,3%)	306
	B- Sangue e órgãos formadores de sangue	101 (72,7%)	38 (27,3%)	139
	C- Sistema cardiovascular	90 (45,5%)	108 (54,5%)	198
	H- Preparações hormonais sistêmicas	54 (38,3%)	87 (61,7%)	141
	L- Agentes antineoplásicos e imunomoduladores	185 (57,3%)	138 (42,7%)	323
	M- Sistema musculoesquelético	41 (80,4%)	10 (19,6%)	51
	N- Sistema nervoso	79 (45,4%)	95 (54,6%)	174
	R- Sistema respiratório	82 (88,2%)	11 (11,8%)	93
	Outros	43 (46,2%)	50 (53,8%)	93
	Total	922 (60,7%)	596 (39,1%)	1.517
Origem da prescrição médica b				
	Serviços privados	224 (59,6%)	152 (40,4%)	376
	Serviços públicos	123 (53,2%)	108 (46,7%)	231
	Serviços filantrópicos	61 (75,3%)	20 (24,7%)	81
	Total	408 (59,3%)	208 (30,2%)	688

Rename: Relação Nacional de Medicamentos Essenciais; ATC: Sistema Anatômico Terapêutico Químico.

a Teste qui-quadrado: p < 0,001.

b Teste qui-quadrado: p < 0,002.

Fonte: Dados da pesquisa, 2019.

Na Tabela 3 , estão listados os 10 medicamentos mais frequentemente solicitados de acordo com sua inclusão na Rename, presença de alternativa terapêutica e, no caso dos que estão presentes em listas oficiais, concordância com os PCDT vigentes. Dos medicamentos não incorporados à política de assistência farmacêutica, a insulina glargina, brometo de tiotrópio, insulinas análogas de ação rápida (lispro e asparte) e medicamentos para tratamento da osteoporose (teriparatida e denosumabe) lideraram os pedidos, não havendo alternativa no SUS para apenas 2 dos 10 medicamentos mais solicitados (bevacizumabe para uso como antineoplásico e para uso intravítreo).

**Tabela 3:** Medicamentos mais solicitados nas ações judiciais de acordo com a presença na Rename, alternativa terapêutica no SUS e concordância com os PCDT. Rio Grande do Norte, 2013–2017.

Medicamentos fora da Rename (n = 922)	n	%	Alternativa terapêutica no SUS	Medicamentos presentes na Rename (n = 596)	n	%	Concordância com PCDT (%)
Insulina glargina	74	8,0	S	Somatropina	53	8,9	100,0
Brometo de tiotrópio	58	6,3	S	Rituximabe	25	4,2	8,0
Enoxaparina	57	6,2	S	Azatioprina	21	3,7	90,5
Insulina lispro	32	3,5	S	Micofenolato de mofetila	18	3,1	0,0
Teriparatida	30	3,3	S	Clopidogrel	16	2,7	12,5
Bevacizumabe (intravítreo)	25	2,7	N	Infliximabe	14	2,3	21,4
Insulina asparte	24	2,6	S	Ácido acetilsalicílico	13	2,2	100,0
Bevacizumabe	21	2,3	N	Goserrelina	13	2,2	23,1
Denosumabe	21	2,3	S	Deferasirox	12	2,0	25,0
Rivaroxabana	19	2,1	S	Mesalazina	12	2,0	83,3

Rename: Relação Nacional de Medicamentos Essenciais; SUS: Sistema Único de Saúde; PCDT: Protocolos Clínicos e Diretrizes Terapêuticas; S: sim; N: não

Fonte: Dados da pesquisa, 2019.

Por fim, dos medicamentos demandados contemplados na política, a somatropina foi o mais solicitado, com 100% de concordância da indicação do requerimento com as diretrizes clínicas do SUS, situação semelhante aos pedidos de azatioprina e mesalazina. Já os demais medicamentos, apesar de estarem na política, foram frequentemente solicitados para indicações não padronizadas nos PCDT, destacando-se o micofenolato de mofetila, que foi demandado judicialmente em 18 ações, nenhuma delas para a indicação padronizada pelo SUS ( Tabela 3 ).

## DISCUSSÃO

No presente estudo, vimos que a maioria dos medicamentos solicitados ao Judiciário não estão incorporados ao SUS, porém há alternativa terapêutica disponível. Existe uma associação entre a classe de medicamentos pleiteada e a sua presença na Rename, havendo uma baixa adesão dos prescritores aos presentes nos componentes da assistência farmacêutica e uma predominância de prescrição de medicamentos fora da Rename por médicos vinculados às instituições filantrópicas e privadas. Mesmo no caso dos fármacos presentes na relação, há prescrição em desacordo com a indicação prevista nos PCDT.

Estudos empíricos que avaliaram o tipo de medicamento requerido mostram que não há um padrão nacional. Existem pesquisas com predominância de pedidos de medicamentos da política pública ^4^^,^^5^^,^^10^^,^^11^ , porém a maioria dos dados empíricos mostram-se semelhantes aos nossos, com os fármacos mais judicializados não sendo contemplados na Rename ^12^^–^^16^ .

Uma hipótese que justifica esse panorama heterogêneo é a de que a judicialização da saúde apresenta diferenças regionais relevantes que se refletem nos sistemas de assistência à saúde e no sistema de justiça. Portanto, nos estados em que o maior número de demandas judiciais é por medicamentos presentes na política nacional, podem haver falhas na gestão da assistência farmacêutica, enquanto naqueles em que os pedidos são predominantemente de fármacos fora de listas oficiais, a política funciona bem, chegando aos tribunais apenas o que não é fornecido ao cidadão porque ainda não está disponível no SUS ^17^ .

Outra hipótese para a judicialização de medicamentos ainda não disponíveis no SUS é que há demora na incorporação de tecnologias, bem como falta de critérios técnicos e transparência no processo de incorporação ^18^ . Porém uma análise que comparou as edições da Rename publicadas entre 2000 e 2014 observou aumento no número de medicamentos nas edições publicadas após 2012 sem a esperada diminuição da judicialização e da pressão pela incorporação tecnológica de produtos inovadores, que, pelo contrário, têm aumentado nos últimos anos ^19^ . A Rename sofreu alterações em seu processo de elaboração, passando de uma lista baseada no conceito de essencialidade nas edições de 1998 a 2010 para uma relação positiva de medicamentos cobertos pelo SUS a partir de 2012, com a publicação da lei nº 12.401/2011 e do decreto 7.508/2011, que regulamentaram o conceito de integralidade no SUS, o que trouxe mudanças para o processo de incorporação de tecnologias ^7^^,^^19^ .

Nossos dados mostraram que a maioria dos medicamentos pleiteados fora da Rename tem alternativa terapêutica no SUS. Por exemplo, um dos mais solicitados foi o brometo de tiotrópio, indicado para o tratamento de asma ou doença pulmonar obstrutiva crônica (DPOC), que conta com a associação de formoterol e budesonida como alternativa terapêutica no SUS, pertencendo ao CEAF. Estudos prévios que avaliaram a proporção de fármacos demandados que têm alternativa terapêutica no SUS mostram prevalências que vão de 41,7% a 96,0%. Esses dados evidenciam que a gestão do SUS não é completamente omissa, pois fornece opções de tratamento para as doenças que justificam os pedidos judiciais ^20^ .

Algumas classes de medicamentos são alvos preferenciais de pedidos de fármacos não incorporados, apesar de não tratarem as doenças mais prevalentes na população. Uma análise dos pedidos de incorporação à Rename de 2012 a 2016 mostrou que os medicamentos das classes dos anti-infecciosos de uso sistêmico, principalmente antivirais para tratamento de HIV/aids e hepatite C, e os indicados para afecções osteomusculares, neoplasias, transtornos mentais e comportamentais e doenças do aparelho respiratório somaram 64,5% dos fármacos incorporados. Esses dados mostram que há um aumento na disponibilização de medicamentos pelo SUS, inclusive de classes que continuam a ser demandadas judicialmente, o que corrobora nossos achados ^21^ .

Ao abandonar o conceito de essencialidade, baseado nas demandas epidemiológicas da população, a Rename tem se tornado mais susceptível às pressões políticas e sociais para incorporação de novas tecnologias, que nem sempre apresentam superioridade terapêutica. Esse é um fato preocupante e que se opõe a diretrizes estabelecidas pela PNM, pois compromete a gestão da AF, a racionalidade no uso de medicamentos e a adesão dos profissionais médicos às listas do SUS ^18^^,^^19^ .

Outra diretriz prioritária estabelecida pela PNM é o uso racional e a segurança dos medicamentos, sendo a prescrição *off label* um indicativo da falta de eficácia e segurança no uso. Nossos dados mostraram que o medicamento mais prescrito para uso *off label* foi o bevacizumabe intravítreo, com 35 solicitações para indicações clínicas diversas, destacando-se a retinopatia diabética, glaucoma e degeneração macular relacionada à idade (DMRI).

O bevacizumabe tem registro na Anvisa com indicação para o tratamento do câncer, porém vem sendo utilizado de forma *off label* para a DMRI. Em vista da intensa judicialização e da falta de opções terapêutica no SUS, em 2016 a Anvisa autorizou seu uso excepcional e temporário por três anos para o tratamento da DMRI, de acordo com regras estabelecidas em um protocolo que prevê o uso intravítreo apenas para DMRI (código H35.3 na 10ᵃ revisão da Classificação Internacional de Doenças) ^22^ . Entretanto, constatamos que, das 35 solicitações judiciais para esse medicamento, apenas cinco apresentaram essa indicação, evidenciando que o SUS tem buscado preencher lacunas nas suas políticas, porém novos usos e indicações acabam por alimentar continuamente a judicialização.

Um dos protagonistas da judicialização da saúde são os profissionais médicos. Estudos sobre a temática têm apontado as relações entre prescritores, indústria farmacêutica e advogados que atuam de forma coordenada, gerando demanda judicial por medicamentos e, assim, pressionando sua incorporação ^6^^,^^23^^,^^24^ . No presente estudo, observou-se que médicos de instituições filantrópicas e de serviços privados de saúde solicitam mais drogas fora da Rename do que os médicos do SUS, apesar de todos os prescritores solicitarem predominante medicamentos não padronizados.

Isso demonstra que os autores provenientes de serviços privados e filantrópicos recorrem ao Judiciário para ter acesso, por meio do SUS, principalmente a medicamentos não incorporados pelo sistema. Ressalta-se que, na nossa amostra, o principal serviço filantrópico que gerou processos judiciais foi um hospital oncológico conveniado ao SUS e que, portanto, já recebe financiamento via Autorização de Procedimento de Alta Complexidade em Oncologia. Estudos empíricos mostram que o principal critério judicial para a concessão de decisão favorável ao litigante são as provas apresentadas pelo autor da ação, geralmente baseadas em um laudo ou receituário médico ^25^^,^^26^ .

Portanto, é preciso ter cautela, sobretudo, em relação à prescrição de medicamentos não pertencentes às listas oficiais de medicamentos ou em desconformidade com os protocolos clínicos oficialmente instituídos. É necessário questionar se o tratamento pretendido tem evidência científica e se é possível substitui-lo por uma alternativa terapêutica disponível na rede pública de saúde, pois do contrário os efeitos nocivos de decisões individuais fragmentadas prejudicarão políticas públicas, que fazem escolhas prioritárias para alocação de recursos escassos. Ignorar políticas públicas acaba por fortalecer situações discriminatórias e tecnologias médicas insustentáveis do ponto de vista científico ^26^^,^^27^ .

Ao analisar os medicamentos mais solicitados não disponíveis no SUS, vemos o predomínio de pedidos de insulinas análogas, tanto de longa quanto de curta ação. Estudos empíricos realizados em outros estados, como São Paulo e Bahia, também encontraram as insulinas análogas como drogas fora da Rename mais solicitadas ^12^^,^^28^^,^^29^ . Dos 10 medicamentos mais pedidos, apenas o bevacizumabe para uso intravítreo e para tratamento oncológico não tem alternativa disponível no SUS.

É interessante observar, ainda, a dinâmica entre a judicialização e a incorporação de tecnologias no SUS. Cinco dos dez medicamentos mais solicitados judicialmente ao Estado no período de estudo, inclusive alguns com parecer contrário à incorporação ao SUS, foram incorporados recentemente, entre 2016 e 2019. O caso das insulinas análogas é didático para essa relação entre judicialização e incorporação, uma vez que, após dois pareceres negativos à incorporação no ano de 2014, a Comissão Nacional de Incorporação de Tecnologias no SUS (Conitec) recomendou a incorporação das insulinas de ação rápida pelo SUS para o tratamento do diabetes mellitus tipo 1 (DM1) em 2017, e, em março de 2019, foi emitido novo parecer favorável à incorporação das insulinas análogas de longa ação para o DM1, mesmo sem novas evidências científicas de superioridade. No parecer, foram descritos como uma questão a ser analisada “os processos de ação judicial desses medicamentos” ^22^ .

Outros medicamentos alvos de demandas judiciais recentemente incorporados foram a enoxaparina para prevenção da trombofilia em gestantes e o já descrito bevacizumabe intravítreo, com uso excepcional aprovado por três anos. Os demais foram avaliados pela Conitec e tiveram parecer contrário à incorporação por não apresentarem eficácia superior à de tecnologias já disponíveis no SUS. Apenas o denosumabe não tem registro de avaliação pela Conitec até o momento ^22^ .

Apesar de a maioria dos medicamentos solicitados pela via judicial não estar presente na Rename, um percentual considerável desses medicamentos já incorporadas pela política também foi pedido, sendo a maioria do CEAF. A procura do Judiciário para obter medicamentos desse componente pode significar falhas na sua gestão, bem como tentativas de driblar os critérios legalmente estabelecidos. Ao analisarmos a concordância entre o medicamento pleiteado e a indicação clínica descrita em laudo médico com o PCDT vigente, vimos possíveis falhas na gestão da assistência farmacêutica nos pedidos pelos medicamentos somatropina, azatioprina, ácido acetilsalicílico e mesalazina, pois a grande maioria das solicitações concordavam com os protocolos. Já para os demais medicamentos solicitados, a maior prevalência foi para indicações clínicas não padronizadas nos protocolos oficiais, a maioria para usos *off label* , Destaca-se o micofenolato de mofetila, para o qual nenhuma das indicações clínicas descritas nas ações judiciais estava de acordo com o PCDT, e em 12 das 18 solicitações foi prescrito para uso *off label* . Observa-se que as ações judiciais geralmente carecem de critérios técnicos comprobatórios, sendo instruídas com laudo e prescrição médica, raras vezes com a presença de exames, e têm tornado a via judicial uma forma mais rápida e com maiores taxas de sucesso em conseguir o medicamento pleiteado, já que esses documentos seriam insuficientes em muitos casos para garantir o acesso pelo SUS ^8^^,^^26^ .

Este estudo apresenta como limitações o fato de retratar uma realidade local; contudo, na falta de estudos multicêntricos e de um panorama nacional da judicialização no acesso a medicamentos, consideramos que pesquisas locais são necessárias para aprofundar o conhecimento sobre o fenômeno. Além disso, foi realizado a partir de dados consultados nos autos processuais disponíveis nos sistemas de gerenciamento dos tribunais, pelas informações obtidas na Sesap/RN, tratando-se de uma amostra de conveniência, já que não se dispõe de um sistema de gerenciamento específico para essas ações judiciais.

A partir da análise dos dados, pode-se concluir que, em muitos casos, o Judiciário gera pontos de ruptura na política pública existente, passando por cima de critérios que foram elaborados para salvaguardar a saúde da população e a gestão mais eficiente dos recursos públicos. Nesse sentido, na intenção de garantir o direito à saúde, regras sanitárias são violadas e a gestão da assistência farmacêutica é dificultada. Ressalta-se que o fornecimento do medicamento não se traduz necessariamente em garantia da saúde do indivíduo. Após a decisão judicial não existe, por parte do Judiciário ou dos serviços de saúde, um acompanhamento oficial da utilização que verifique as condições de uso, a evolução ou o alcance das metas terapêuticas pretendidas pelo prescritor, desconhecendo-se os desfechos sanitários para a saúde dos pacientes. Desse modo, novas pesquisas devem ser direcionadas para essa análise.
